# Supplementary material for: The interactome of intact mitochondria by cross-linking mass spectrometry provides evidence for coexisting respiratory supercomplexes
Source: Mol Cell Proteomics. 2017 Dec 8;17(2):216–32. doi: 10.1074/mcp.RA117.000470 (PMC5795388; doi:10.1074/mcp.RA117.000470)
Supplement: Supplemental Data [file supp_RA117.000470_133922_0_supp_23978_fzffwf.zip › spectra_annotation/mito_spectra_annotation/490-1-1-1-136-1.pdf]

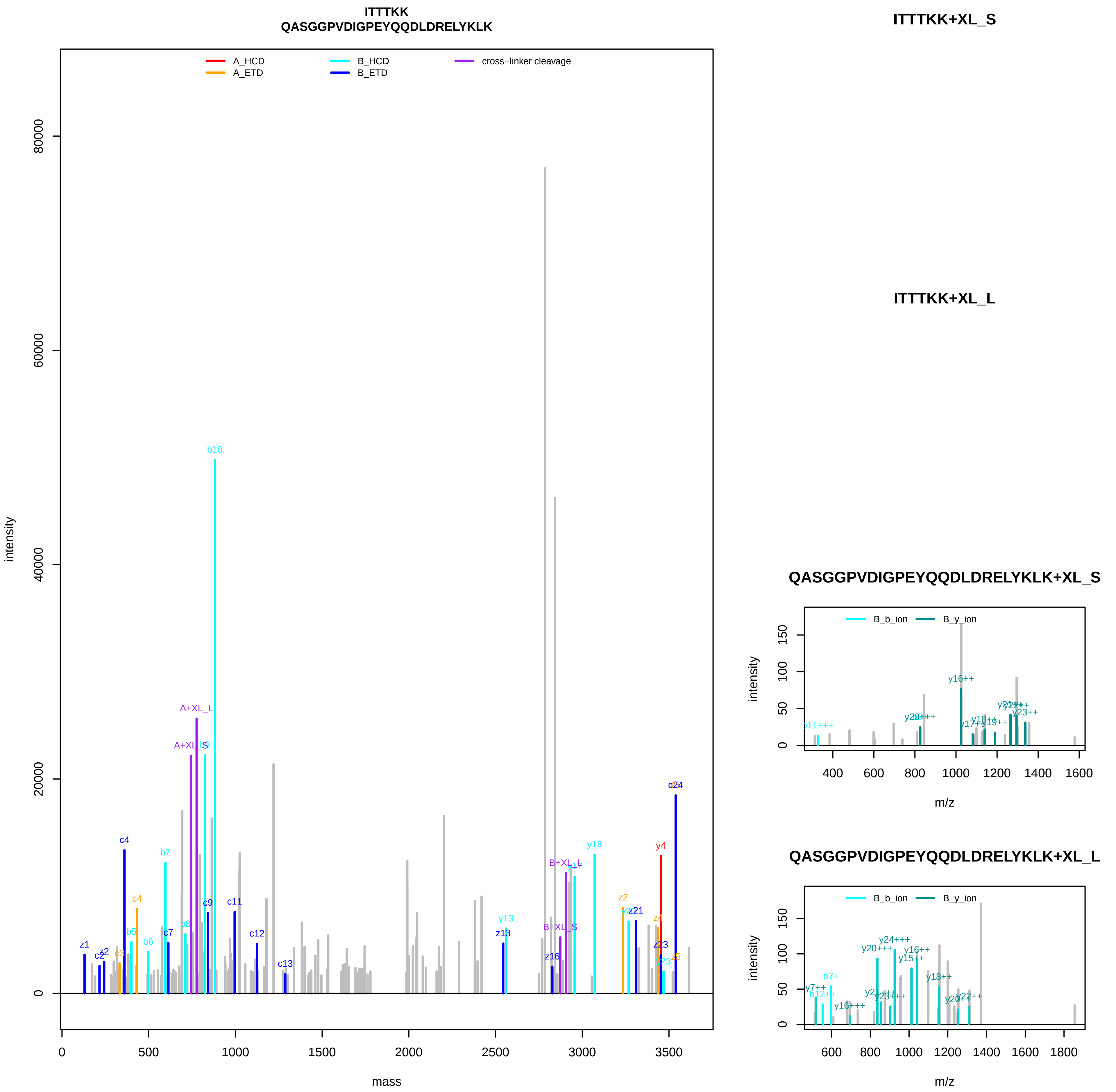
ITTTKK
QASGGPVDIGPEYQQDLDRELYKLK
A_HCD
A_ETD
B_HCD
B_ETD
cross−linker cleavage
intensity
mass
0
20000
40000
60000
80000
0
500
1000
1500
2000
2500
3000
3500
b10
A+XL_L
A+XL_S
c4
b7
c9
c11
c12
c13
z1
c2
z2
c3
b5
b6
c7
b8
y13
z13
z16
B+XL_L
B+XL_S
y17
y18
z2
z21
z4
z23
y4
c24
z5
ITTTKK+XL_S
ITTTKK+XL_L
QASGGPVDIGPEYQQDLDRELYKLK+XL_S
B_b_ion
B_y_ion
m/z
y16++
y20+++
y17++
y18++
y19++
y22++
y23++
0
50
100
150
400
600
800
1000
1200
1400
1600
QASGGPVDIGPEYQQDLDRELYKLK+XL_L
B_b_ion
B_y_ion
m/z
y24+++
y20+++
y16++
y15++
b7+
y7++
b12++
y16+++
y21+++
y23+++
y18++
y22++
0
50
100
150
600
800
1000
1200
1400
1600
1800
